# Influenza Virus Specific CD8^+^ T Cells Exacerbate Infection Following High Dose Influenza Challenge of Aged Mice

**DOI:** 10.1155/2013/876314

**Published:** 2013-09-26

**Authors:** E. M. Parzych, L. J. DiMenna, B. P. Latimer, J. C. Small, S. Kannan, B. Manson, M. O. Lasaro, E. J. Wherry, H. C. Ertl

**Affiliations:** ^1^Wistar Institute, Philadelphia, PA 19104, USA; ^2^Drexel University, Philadelphia, PA 19104, USA; ^3^Columbia University, New York, NY 10027, USA; ^4^Graduate Group of the University of Pennsylvania, Philadelphia, PA 19104, USA; ^5^Cheyney University, Thornbury Township, PA 19139, USA; ^6^Boehringer Ingelheim GMBH, Ridgefield, CT D6877, USA; ^7^Department of Microbiology and Institute for Immunology, University of Pennsylvania, Philadelphia, PA 19104, USA

## Abstract

Influenza viruses cause severe illnesses and death, mainly in the aged population. Protection afforded by licensed vaccines through subtype-specific neutralizing antibodies is incomplete, especially when the vaccine antigens fail to closely match those of the circulating viral strains. Efforts are underway to generate a so-called universal influenza vaccine expressing conserved viral sequences that induce broad protection to multiple strains of influenza virus through the induction of CD8^+^ T cells. Here we assess the effect of a potent antiviral CD8^+^ T cell response on influenza virus infection of young and aged mice. Our results show that CD8^+^ T cell-inducing vaccines can provide some protection to young mice, but they exacerbate influenza virus-associated disease in aged mice, causing extensive lung pathology and death.

## 1. Introduction

The elderly constitute an increasingly large proportion of the human population, posing major challenges to local health care systems worldwide. The general health status varies widely among older individuals [1], ranging from fully functional to functionally disabled individuals with multiple comorbidities. Influenza is one of the top 10 causes of death in older adults, causing in the US in excess of 44,000 deaths on average each year [2, 3]. Underlying chronic diseases dramatically increase the risk of serious complications of influenza virus infection [4, 5]. A trivalent inactivated vaccine for influenza consisting of two strains of influenza A and one strain of influenza B virus is approved for use in the elderly but provides only 30–40% protection in humans above the age of 65 [4, 5]. 

Current influenza vaccines induce protection through strain-specific neutralizing antibodies. The virus mutates rapidly and antigenic variations of the two-surface proteins, the hemagglutinin (HA) and the neuraminidase (NA), allow for the development of antigenic drift strains that partially evade protective humoral immune responses. Therefore, vaccine compositions have to be reformulated annually to incorporate antigenic drift strains. Rearrangements of the segmented viral genes, especially those encoding HA and NA, result in more dramatic changes, or antigenic shifts, and most pandemics are caused by such new strains of influenza virus. To prevent catastrophic outcomes of influenza virus pandemics with newly evolved strains, efforts are underway to develop so-called universal flu vaccines based on viral sequences that are highly conserved across heterologous strains. Such sequences include the stalk domain of HA, which induces neutralizing antibodies that, unlike those against the outer loops, do not agglutinate red blood cells and cross-react between several strains of influenza A virus [6–8], the ectodomain of matrix 2 (M2e) protein, which elicits protective non-neutralizing antibodies [9, 10] and the internal nucleoprotein (NP) and matrix protein that induce potent CD8^+^ T cell responses [11, 12], which have been linked to resistance against influenza A virus infection in humans [13]. A broadly efficacious universal influenza vaccine should aim to elicit a broad range of cross-reactive immune responses to all of these conserved viral sequences.

Here we tested the effect of NP-specific CD8^+^ T cells on influenza A virus challenge in young and aged mice. As we reported previously, aging moderately affects kinetics and magnitude of primary and secondary T cell responses to vaccination or infection [14, 15] which, in part, reflects a loss of naïve virus-specific precursors in the aged [16]. Here we tested the effect of vaccination with a CD8^+^ T cell inducing vaccine to influenza virus in a series of experiments in young and aged mice as detailed in Table 1. Results demonstrate that immunization with a CD8^+^ T cell-inducing vaccine followed by a sublethal infection elicits potent CD8^+^ T cell responses in young as well as aged mice. Such CD8^+^ T cells, especially if present at very high frequencies following prime-boost regimens, may contribute to protection in young mice but exacerbate disease in the aged. 

## 2. Results

### 2.1. Vaccine-Induced NP-Specific CD8^+^ T Cell Responses in Blood

**Figure 1:**
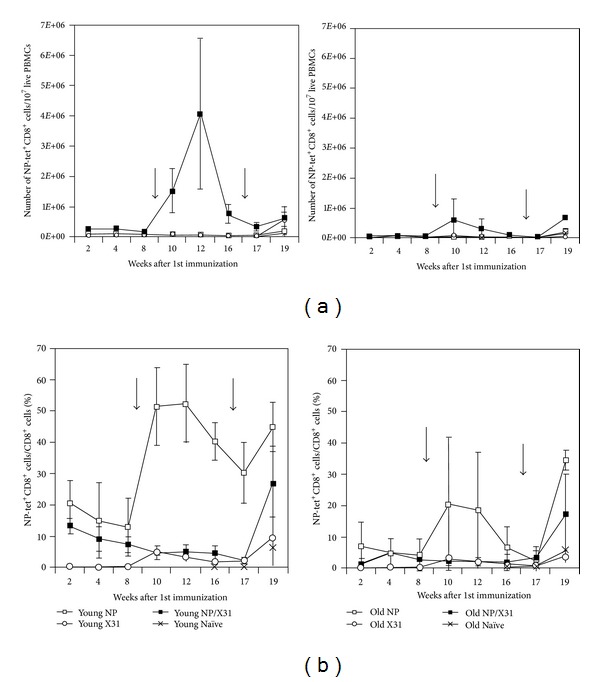
Vaccine-induced NP-specific CD8^+^ T cell responses in young (left) and aged C57Bl/5 female mice (right) in response to vaccination. Groups of young mice (6–8 weeks, *n* = 20) and aged mice (>18 months, *n* = 23) were vaccinated with 10^10^ vp of AdC68NP, given IM. They were bled 2, 4, 6, and 8 weeks after vaccination. PBMCs were isolated and stained with an NP-specific tetramer and antibodies to CD8 to identify the frequency of NP-specific CD8^+^ T cells. A portion of AdC68NP-primed mice were then boosted with 0.8 × 10^5^ TCID_50_ of A/X31, along with age-matched groups of previously naïve mice.

Young (8–12 weeks) and old (20–22 months) C57Bl/6 mice were vaccinated with 10^10^ virus particles (vp) of an E1-deleted adenovirus vector of chimpanzee origin expressing NP of influenza A/PR8 virus (AdC68NP) given intramuscularly (i.m.) or with A/X31, an attenuated H3N2 influenza A virus, given at ~1 × 10^5^ TCID_50_ intranasally (i.n.). Other mice were primed with AdC68NP and then boosted with A/X31 using the same routes and doses. Mice were challenged 4 months after priming or 2 months after boosting with 3 LD_50_ of A/PR8 virus, a mouse virulent H1N1 influenza A virus strain. Mice were bled periodically after immunizations and challenge to determine frequencies and numbers of NP-specific CD8^+^ T cells in blood by staining with an NP epitope-specific tetramer. Both aged and young mice showed similar patterns of responses, although responses were lower overall in aged mice (Figures 1(a), 1(b)). A/X31 induced modest responses. NP-specific CD8^+^ T cell numbers and frequencies were higher after AdC68NP vaccination. Following the boost, both young and aged mice showed increases in NP-specific CD8^+^ T cell frequencies as well as numbers. While peak frequencies were reached in young mice by day 14 after the boost, numbers peaked later in young but not aged mice reflecting increases of circulating CD8^+^ T cells in the former. After A/PR8 challenge, NP-specific CD8^+^ T cell frequencies and numbers expanded by day 20 in all of the vaccine groups and in unvaccinated control mice. Responses were extremely variable in aged mice. (Figure 1(a) shows numbers of NP-specific CD8^+^ T cells/10^7^ CD8^+^ T cells over time). (Figure 1(b) shows frequencies of NP-specific CD8^+^ T cells over time. Closed squares: mice that only received the initial AdC68NP vaccination, without boost (young: *n* = 15, aged: *n* = 13); open circles: mice that received the A/X31 boost only (young: = 5, aged: *n* = 10); open squares: mice that received the prime/boost regimen (young: *n* = 4, aged: *n* = 13). Mice were then bled 2, 4, 6, and 8 weeks after the boost, and frequencies of NP-specific CD8^+^ T cells in blood were determined. Mice were challenged 2 months after the boost with 3LD_50_ A/PR8, along with additional age-matched controls (represented by (X), young: *n* = 25, aged: *n* = 33). Arrows represent the boosting and challenge events, respectively. Final responses were assessed 20 days after challenge. Graphs show average numbers or frequencies of NP-specific CD8^+^ T cells ± SD over weeks following the initial immunization).

### 2.2. Numbers of NP-Specific CD8^+^ T Cells in Blood and Tissues before and after A/PR8 Challenge

**Figure 2:**
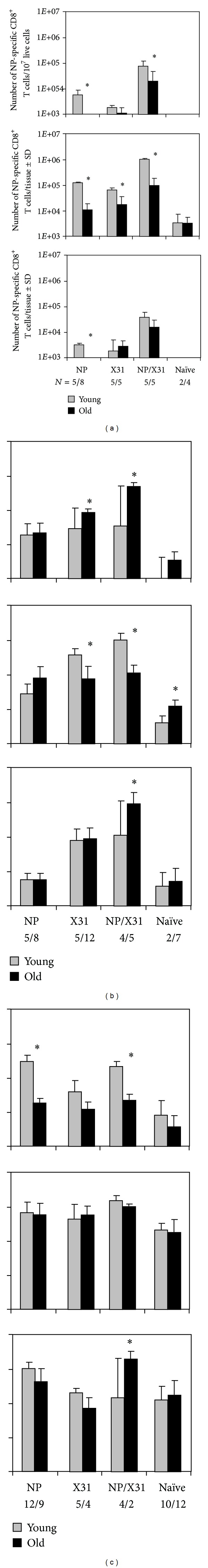
Vaccine-induced numbers of NP-specific CD8^+^ T cells in the blood, spleen, and lung. Groups of young and aged mice were vaccinated with 10^10^ vp of AdC68NP, given IM. A portion of these primed mice, along with age-matched groups of naïve mice, were subsequently boosted at 2 months after priming with 0.8 × 10^5^ TCID_50_ of A/X31, given IN. 2 months after the boost, cohorts were euthanized before or after challenge with 3LD_50_ A/PR8. Lymphocytes were harvested from the blood, spleen, and lungs of these mice and underwent tetramer and CD8 staining to determine the total number of tetramer^+^CD8^+^ cells in each compartment. Graphs show numbers of NP-specific CD8^+^ T cells over 10^7^ live PBMCs (upper row) or per all live mononuclear cells from spleens (middle rows) or lungs (low rows). The bars reflect group averages ± SD; gray bars represent young mice and black bars represent aged mice. Asterisks indicate significant differences between groups at the *P* ≤ 0.05 level.

Frequencies and numbers of circulating CD8^+^ T cells may not be informative, as protection would be expected to rely on T cells that migrate to the site of infection. We therefore determined absolute numbers of NP-specific CD8^+^ T cells in lungs and spleens and numbers per 10^7^ live lymphoid cells in blood before and after challenge of vaccinated mice. Before challenge, numbers of NP-specific CD8^+^ T cells were low in blood of young and aged mice that had been immunized once with either AdC68NP or A/X31. Mice that received the prime boost regimen had increased numbers of NP-specific CD8^+^ T cells and, again, they were significantly higher in young mice than in aged mice. In spleens, NP-specific CD8^+^ T cell numbers were higher in young mice following all immunization regimens. In lungs, numbers were comparable in most groups between young and aged mice. (Figure 2(a) shows results for cells were harvested 2 months following vaccination without further A/PR8 challenge: AdC68NP primed only, A/X31 only, AdC68NP followed by A/X31, and naïve). By 5 days after A/PR8 challenge, numbers of NP-specific CD8^+^ T cells markedly increased in blood and more modestly in spleens of most groups. The most pronounced increases were observed in lungs; numbers were similar between the two age cohorts except for the prime boost group, where significantly higher numbers of NP-specific CD8^+^ T cells were found in aged mice. (Figure 2(a) shows results for cells harvested 5 days following A/PR8 challenge in all groups: AdC68NP primed only, X31 boost only, AdC68NP followed by A/X31, and naïve) By day 20 after A/PR8 infection, numbers of NP-specific CD8^+^ T cells increased further in blood of AdC68NP vaccinated young mice and in naïve mice of both age groups and remained stable or decreased as compared to day 5 in the aged. In spleens and lungs, numbers of NP-specific CD8^+^ T cells also increased in most groups. (Figure 2(c) shows results for cells harvested 20 days following A/PR8 challenge in all groups: AdC68NP primed only, A/X31 boost alone, NP/X31, and naïve. Numbers of mice per group are indicated below the graphs).

### 2.3. Cytokine Production by NP-Specific CD8^+^ T Cells from Young and Aged Mice

**Figure 3:**
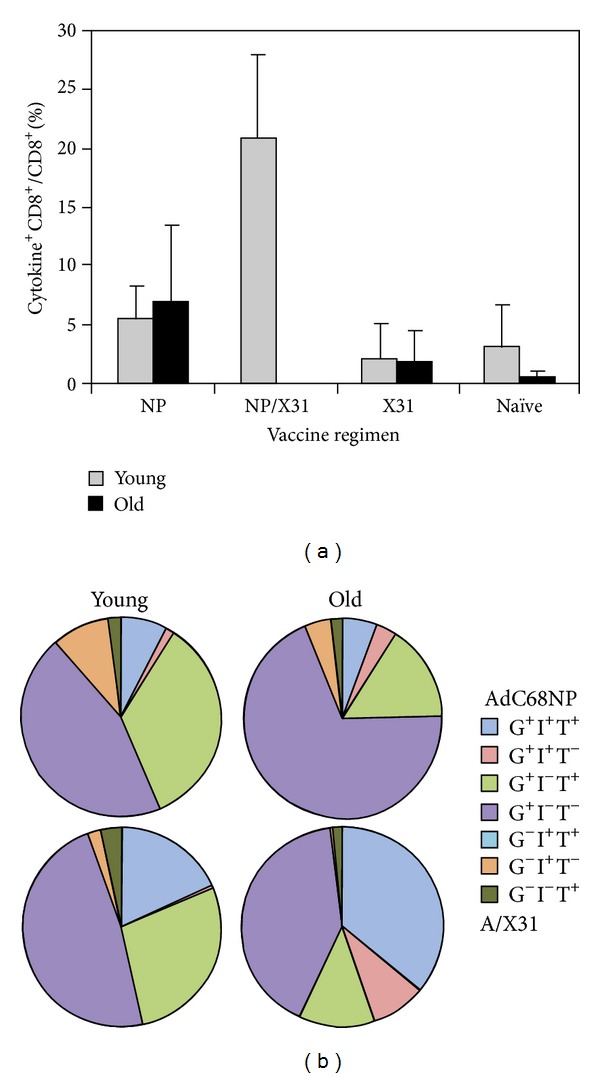
Cytokine production by vaccine-induced NP-specific CD8^+^ T cells. Groups of young and aged mice were either vaccinated with 10^10^ vps of AdC68NP only (young and aged: *n* = 4 each) or were then subsequently boosted 8 weeks later with 0.8 × 10^5^ TCID_50_ of A/X31 (young: *n* = 4, aged not tested as all died following infection). Additional age-matched groups were added that received only A/X31 boost (young and aged: *n* = 4 each). All mice were then challenged with 3LD_50_ A/PR8 2 months following the boost, along with age-matched control groups (young: *n* = 5, aged: *n* = 3). At day 20 following the challenge, splenocytes were isolated, and an ICS was conducted to detect production of IFN-*γ* (G), IL-2 (I), and TNF-*α* (T).

We tested splenocytes harvested 20 days after A/PR8 challenge by intracellular cytokine staining (ICS) for IFN-*γ*, TNF-*α*, and IL-2. Frequencies of cytokine producing NP-specific CD8^+^ T cells were similar in young and aged mice (Figure 3(a) shows sum of frequency of cytokine producing NP-specific CD8^+^ T cells in response to stimulation with NP peptide) although they were markedly lower than those obtained with the MHC class I tetramer. In AdC68NP-immunized mice, NP-specific CD8^+^ T cells from old mice produced mainly IFN-*γ* only; those from young mice had a higher portion of cells that produced IFN-*γ* together with TNF-*α*.  Figure 3(b) show percentages of the 7 different combinations of cytokine production profiles.) In A/X31 immune mice, observed differences between aged and young mice were subtle.

### 2.4. Vaccine-Induced Protection upon A/PR8 Challenge

Vaccine-induced protection was assessed by determining survival of mice following challenge with 3LD_50_ of A/PR8 virus. Mice were either vaccinated with 10^10^ vps AdC68NP only (young: *n* = 15, aged: *n* = 13) or additionally received a subsequent boost of 0.8 × 10^5^ TCID_50_ of A/X31 two months after the priming (young: *n* = 4, aged: *n* = 8). Additional age-matched groups were added at the time of boosting and received A/X31 (young: *n* = 5, aged: *n* = 10). All mice, along with age-matched control groups (young: *n* = 25, aged: *n* = 38), were challenged with 3LD_50_ of A/PR8. Mice were monitored daily and euthanized once they lost ≥30% of their original weight, although aged mice commonly died before then with minimal weight loss. Graphs show survival over days after infection. The following differences between vaccinated and naïve mice were statistically significant (log-rank test): old-AdC68NP: *P* = 0.0005, old-A/X31: *P* = 0.0011, old AdC68NP/A/X31: *P* = 0.0049; young-AdC68NP *P* = 0.021.) In both age cohorts, approximately 40% of naïve mice survived the challenge. Young mice vaccinated with A/X31 alone or AdC68NP followed by A/X31 were completely protected from death. AdC68NP alone provided only marginal protection to young and aged mice. Aged mice immunized with A/X31 showed a delay in mortality. Surprisingly, most aged mice that received the AdC68NP vaccine followed by an A/X31 boost very rapidly succumbed to the infection. We used two different prime boost regimens for aged mice. In one regimen, mice were vaccinated at the age of ~3 months and then boosted once they were 22 months old. In the other regimen, mice were vaccinated with AdC68NP at 20 months of age and they were also boosted at 22 months of age. Results for T cell responses were similar in both groups and are therefore shown together in the graphs. It was noteworthy that the only two old mice in the prime boost groups that survived the challenge were both primed at 20 months of age. 

**Figure 4:**
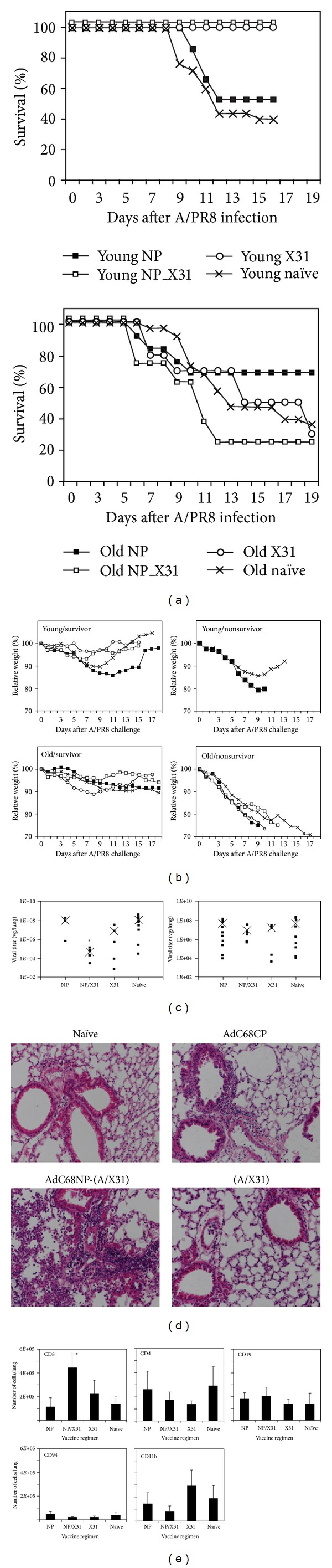
(a) Survival of young and aged mice following A/PR8 challenge. (b) Weight loss following A/PR8 challenge. (c) Lung viral titers in lungs at day 5 after A/PR8 challenge. (d) Lung histology. (e) Lung infiltrates.

In young mice, A/X31 vaccination protected against weight loss; young mice that received AdC68NP alone or AdC68NP followed by A/X31 initially lost weight at a similar rate as control mice. By day 5 after infection, the prime boost group of young mice regained weight 2-3 days prior to control mice. Weight gain was delayed in the AdC68NP only group. In young mice that died following infection, the AdC68NP vaccinated mice showed more pronounced weight loss compared to control mice. Aged mice on average lost less weight compared to young mice. In the surviving cohort of aged mice, the two that received the prime-boost lost the least weight, while aged A/X31 vaccinated mice showed the most pronounced initial weight loss. Unlike young mice, old mice failed to regain weight with the exception of those that were only immunized with A/X31. (Figure 4(b) shows weight loss of the same mice as shown in Figure 4(a). The upper panels show average weight loss, by group, in the aged mice that survived (left) or succumbed (right) to the challenge. The same information is provided for young mice in the lower panels. Closed squares: AdC68NP prime only, open squares: AdC68NP followed by A/X31, circles: A/X31 only, X: controls. Graphs show relative weight loss over days after infection. Differences in weight loss at the time of maximal weight loss were significant for the following vaccine groups in comparison to naïve age-matched mice (*t*-test with Bonferroni correction): old-AdC68NP: *P* = 0.0050, old-A/X31: *P* = 0.0213, young-A/X31: *P* = 0.021, young-AdC68NP/A/X31: *P* = 0.017).

We measured lung virus titers on day 5 following A/PR8 challenge. Only young mice that were vaccinated with AdC68NP followed by A/X31 showed significantly reduced viral titers as compared to the naïve controls. Aged unvaccinated and vaccinated mice had comparable titers. (Groups of young (top) and aged mice (bottom) received a single immunization with 10^10^ vps AdC86NP only (young: *n* = 5, aged: *n* = 15); additional mice were boosted 2 months later with 1 × 10^5^ TCID_50_ A/X31 (both age groups: *n* = 5) or received only the A/X31 immunization (young: *n* = 5, aged: *n* = 7.) All mice, along with unimmunized control mice (young: *n* = 9, aged: *n* = 11), were challenged with 3LD_50_ A/PR8 and then sacrificed on day 5 after challenge. Lung viral titers were determined by quantitative RT-PCR. Data shown in Figure 4(b) are expressed as viral genomes per gram of tissue. Each data point represents an individual mouse while (X) indicates the group average. Differences in viral and titers between naïve and age-matched vaccinated mice were significant for the following group: young AdC68NP/A/X31: *P* = 0.048).

We examined thin HE-stained lung sections of aged mice that had been challenged 5 days previously with 3LD_50_ of A/PR8 virus. (Figure 4(d) shows hematoxylin and eosin-stained lung sections of aged mice, from each of the three experimental regimens (plus control), harvested on day 5 after challenge with 3LD_50_ of A/PR8.) Control aged mice and aged mice vaccinated with A/X31 or AdC68NP presented with similar lung pathology, which consisted mainly of perivascular infiltrates and some limited interstitial inflammation. In contrast, aged mice that received the prime boost regimen had extensive interstitial infiltrates that, in some mice, afflicted more than 50% of the lung parenchyma. In contrast, young mice that received the prime-boost regimen only showed minor pathology (data not shown). 

To determine the cell types that caused the severe inflammation in aged mice, we isolated mononuclear cells on day 5 after A/PR8 infection from lungs of aged mice and stained cells with lineage defining markers, that is, CD8 and CD4 for T cell subsets, CD19 for B cells, CD11b for monocytes, Gr-1 for neutrophils and CD94 for NK cells. Only CD8^+^ T cells were significantly increased in the lungs of aged mice that had received the prime-boost regimen.  Figure 4(e) shows distribution of mononuclear cell subpopulations as found in the lung of aged mice that received either a single vaccination of AdC68NP (*n* = 7) or A/X31 (alone (*n* = 4) or an AdC68NP-A/X31 prime/boost regimen (*n* = 5) or were left naïve (*n* = 7). Lungs were harvested 5 days following challenge with 3LD_50_ A/PR8. Mononuclear cells were isolated and stained with fluorochrome-labeled antibodies to several cell surface markers, analyzed by flow cytometry and then FlowJo software. After gating on a cell population encompassing lymphocytes and monocytes, and then onto singlets, populations were identified sequentially as follows: CD8^+^CD4^−^, CD8^−^CD4^+^, and CD8^−^CD4^−^. The CD8^−^CD4^−^ population was then gated onto CD19^+^ and CD19^−^ cells. The CD19^−^ population was gated onto CD94^+^ and CD94^−^ cells. CD19^−^ cells were then gated on CD11b^+^ cells. The graph shows the average total number of each subset within the lung compartment. The asterisks indicate statistical significance (*t*-test, Bonferroni corrected *P* ≤ 0.05), when compared to the corresponding naïve group. 

**Figure 5:**
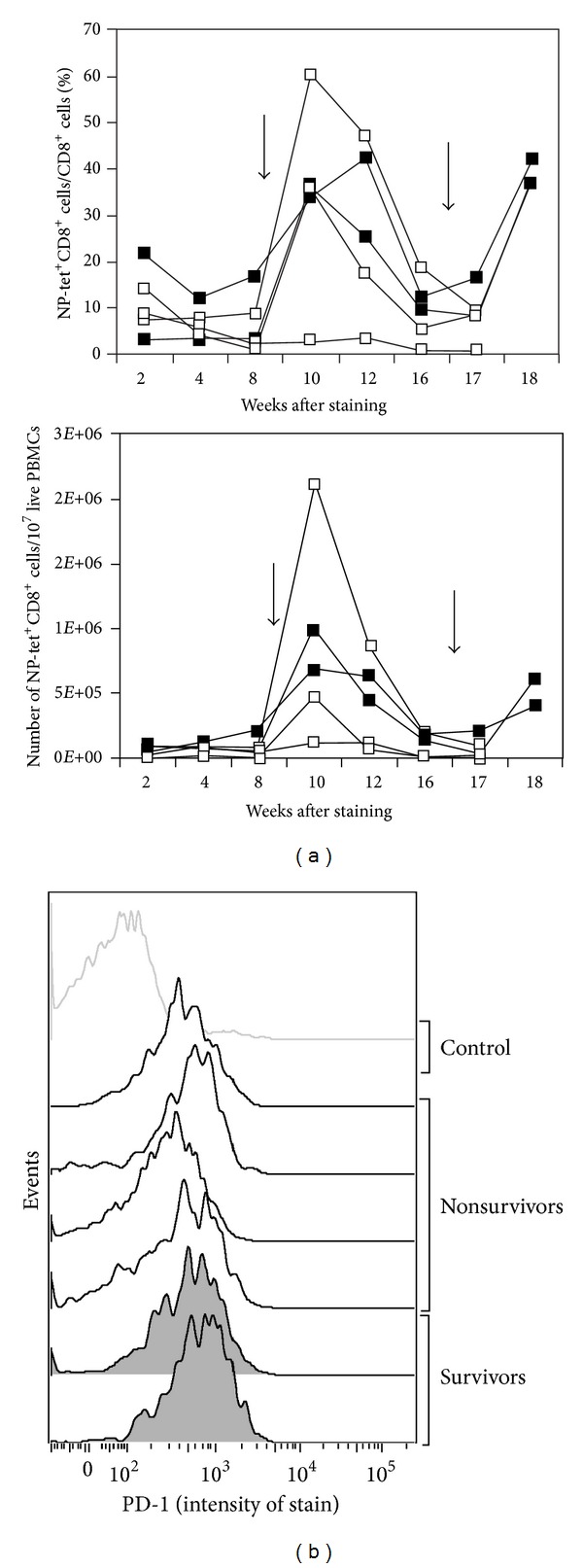
Comparison of CD8^+^ T cell frequencies and phenotypes between protected and nonprotected aged mice.

Two aged mice that received a prime-boost regimen survived the A/PR8 infection with only little to moderate weight loss, while all of the other aged mice that received this vaccine regimen in this and other experiments lost weight rapidly and died (Figures 4(a), 4(b)). To determine if the two survivors failed to become infected or if any parameters of their T cell response predicted their relative resistance to challenge, we compared frequencies and numbers of NP-specific CD8^+^ T cells of surviving versus nonsurviving mice in blood. As shown in Figure 5(a), the two surviving mice had average NP-specific CD8^+^ T cell responses. Both showed expansion of NP-specific CD8^+^ T cells in blood following challenge, indicating that they had become infected. (Figure 5(a) shows frequencies (top) and numbers per 10^7^ PBMCs (bottom) of NP-specific CD8^+^ T cells over time in aged mice that received the AdC68NP prime-A/X31 boost regimen. Mice were challenged with 3LD_50_ A/PR8 2 months after the boost. The closed squares represent the mice that survived for 20 days following the challenge (*n* = 2), while the open squares reflect mice that did not survive the challenge (*n* = 3). The arrows indicate the boosting and challenging events, resp.) Testing for surface markers of NP-specific CD8^+^ T cells harvested from blood 2 days prior to challenge showed similar patterns for a number of markers such as CD44, CD62L, and CD127. Only PD-1, a marker for T cell activation/exhaustion appeared to correlate with protection; specifically, NP-specific CD8^+^ T cells from mice that survived expressed higher levels of PD-1 (mean fluorescent intensity [MFI] of 648) compared to those that succumbed the infection (MFI of 400). Although this difference was statistically significant (*P* = 0.047 by student *t*-test), the increase in surface expression levels was subtle.  Figure 5(b) PD-1 expression on the surface of NP-specific or naïve CD8^+^ T cells in the blood of these mice two days prior to challenge (8 weeks after the boost). The shaded areas represent the survivors, the black lines show the nonsurvivors, and the light gray histogram shows expression levels on naïve CD8^+^ T cells (tetramer-CD44^low^). With the caveat that these results are based on small numbers of mice, one could speculate that the elevated levels of PD-1 suggest an impairment of CD8^+^ T cell functions, which reduced CD8^+^ T cell-mediated immunopathology and thus allowed the aged mice to survive the challenge.

## 3. Discussion

Improved influenza vaccines that provide broad and sustained protection against a wide range of antigenically distinct influenza A viruses are in demand. Most preclinical studies on so-called universal influenza A virus vaccines have focused on young mice despite the fact that, in humans, influenza A virus-related mortality mainly affects the aged. Immune responses become impaired during aging, resulting in an increased susceptibility to infectious agents and an inability to mount protective immune responses after vaccination. Immunosenescence affects multiple aspects of the immune system, as has been demonstrated in aged humans and mice. B cell lymphopoiesis is reduced with aging, leading to a decline of naïve B cells and an increase of antigen-experienced B cells with an extended lifespan. Primary B cell responses in the elderly are commonly both low and short-lived, resulting in antibodies with low affinity [17]. Formation of germinal centers is decreased [18, 19]. Autoantibodies are more common and the B cell repertoire becomes more restricted [17]. The E2A-encoded transcription factor E47 is downregulated in old splenic B cells, which causes a reduction in the activation-induced cytidine deaminase, needed for class switch recombination and immunoglobulin (Ig) somatic hypermutation [18]. Some of the defects of B cell responses are secondary to an age-related decline of helper functions from CD4^+^ T cells, which show reduced expression of critical costimulatory receptors that are essential for activation of B cells, germinal center formation and rearrangement, and hypermutation of Ig genes [19–21]. T cells show clonal senescence; their potential for expansion is decreased and their ability to produce certain cytokines or to respond to cytokines may decrease. The proportion of T cells with a memory cell phenotype increases, while numbers of naïve T cells decrease. Expression of activating costimulatory molecules such as CD40 ligand and CD28 decreases [19, 22], while inhibitory pathways increase [15, 16]. Stimulation with new antigens or previously encountered antigen results in CD8^+^ T cell responses that are delayed and show defects in transition into memory. Upon aging the T cell repertoire loses diversity [23, 24]. In summary, although numerous studies have described defects that accumulate in the immune system of the aged, the mechanisms underlying these defects are not yet clear. We recently reported on an AdC68-based universal influenza vaccine, which expresses M2e peptides for induction of antibodies linked to NP for stimulation of T cells. This vaccine induces protective immunity in young mice, which requires stimulation of both antibody and CD8^+^ T cell responses; the vaccine lacked efficacy in aged mice [25]. 

We undertook the current study to further address the role of influenza virus-specific CD8^+^ T cells in providing resistance to influenza A virus infection in aged mice.Our results show that aged mice develop NP-specific CD8^+^ T cells, which expand upon subsequent infection with A/X31 virus. T cell responses in the aged are lower, less sustained, and more variable compared to those in young mice. We used two prime boost regimens; one in which mice were primed at a young age and another in which mice were primed at an old age, assuming that a recall response of memory T cells that had been generated prior to immunosenescence would result in superior CD8^+^ T cell responses, as has been reported previously [26]. Regarding the magnitude of responses, this could not be confirmed. We observed an age-related difference in T cell homing; aged mice, compared to young mice, had relatively higher frequencies of NP-specific CD8^+^ T cells in blood and lung than spleen, potentially suggesting that T cells remained more activated. Differences in T cell functionality regarding cytokine production could be observed but were overall subtle and may have been a reflection of decreases in T cell receptor (TcR) signaling due to increases in coinhibitors on aged T cells [16] or reductions in expression of peroxisome proliferator-activated receptor (PPAR)*α* expression [27], which in turn inhibits expression of T-bet, a transcription factor that promotes synthesis of IFN-*γ* and inhibits production of IL-2. Differential changes in cytokine production induced by the AdC68NP vaccine given intramuscularly as compared to A/X31, which was applied to the nostrils, may reflect a slower rate of age-related decline of cells of the mucosal immune system [28]. The most marked difference was seen in vaccine-induced protection against A/PR8 challenge. Young mice that were vaccinated with A/X31 alone or primed with AdC68NP and then boosted with A/X31 were completely protected against death following A/PR8 challenge. In addition, young mice that received the prime-boost regimen had significantly reduced viral titers by day 5 following infection. Protection induced by A/X31 may be mediated by several immune mechanisms including CD8^+^ T and B cells to conserved epitopes present on different proteins of the virus. AdC68NP priming increases primarily CD8^+^ T cell responses to NP. We therefore assume that the accelerated viral clearance in young mice that received the prime boost regimen could mainly be attributed to the activity of NP-specific CD8^+^ T cells. Different immune mechanisms can provide protection against influenza virus infection or accelerate clearance of virus. Neutralizing antibodies cannot only prevent or reduce disease if present prior to infection but they can also contribute to viral clearance in animals that lack CD8^+^ T cells [28]. In our experiments, the pronounced protection of young mice that had been immunized with A/X31 with or without prior injection of AdC68NP against A/PR8 challenge may have reflected an accelerated production of A/PR8 neutralizing antibodies due to preexisting T cell helper cells. We tested vaccinated in comparison to naïve mice for HA-specific antibodies following challenge and failed to observe accelerated increases in such antibodies (data not shown). Although we cannot rule out that immune mechanisms other than NP-specific CD8^+^ T cells may have contributed to protection, our data do not support the notion that HA-specific antibodies played a dominant role. Antibodies to NP have been reported to contribute to viral clearance [29]. We tested young and aged mice immunized with the AdC68NP vaccine for antibodies to NP; while young mice mounted a robust response, most of the aged mice failed to generate sustained antibody titers to NP (data not shown). Since the AdC68NP vaccine induced some protection in both age groups, we doubt that NP-specific antibodies contributed to protection although we cannot rule out that they, together with other immune mechanisms, may have played a role in protection of young mice upon A/X31 or AdC68NP/A/X31 immunization. Additionally, antibodies to M2e, which could have been induced at low levels in response to A/X31 immunization [30], may have contributed to protection in young mice. A number of publications implicated a role for CD4^+^ T cells in heterotypic protection against influenza virus challenges [31–33] and again such T cells could have been induced by either of the vaccines. The role of CD8^+^ T cell-inducing vaccines in providing protection against influenza A virus continues to be debated; some investigators reported solid protection in young mice [11, 34–36], while others showed complete lack of efficacy [37]. CD8^+^ T cells can mediate viral clearance by direct lysis of infected cells or by secretion of antiviral cytokines such as IFN-*γ* or chemokines that recruit other antiviral effectors. A recent report showed that the predominant type of CD8^+^ T cell function within influenza A virus-infected lungs is dictated by the target cells [38]. Infected respiratory epithelial cells elicit mainly a lytic reaction while lung residing antigen-presenting cells trigger cytokine release, presumably by providing stronger TcR signaling through simultaneous engagement of costimulators. In humans, high frequencies of antigen-specific CD8^+^ T cells were reported to reduce the severity of infections [13]. In the last pandemic of 2009, mainly the young to middle aged became infected while the elderly showed relative resistance [39]. This was most likely linked to cross-reactive antibodies induced by strains that circulated prior to 1957 [40] although it has been argued that cross-reactive CD8^+^ T cells may also have provided some protection [41]. 

In aged mice, priming with AdC68NP followed by a booster immunization with A/X31 also markedly enhanced frequencies and numbers of NP-specific CD8^+^ T cells in blood and lungs, which further increased in lungs following A/PR8 challenge. Nevertheless, aged mice were not protected upon sequential vaccination but, instead, died rapidly following challenge. Analyses of lung-infiltrating cells 5 days after challenge showed a very pronounced accumulation of CD8^+^ T cells in prime-boosted aged mice. In most compartment using most of the different vaccine regimens, younger mice developed higher frequencies and numbers of NP-specific CD8^+^ T cells compared to aged mice. Upon the AdC68NP-A/X31 prime boost regimen followed by A/PR8 challenge aged mice developed significantly higher numbers of NP-specific CD8^+^ T cells in blood and lungs compared to young mice. We assume that these massive numbers of NP-specific CD8^+^ T cells within the infected lungs caused a catastrophic destruction of functional tissue. It is well documented that immunopathology is linked to morbidity and mortality following influenza virus infection and a number of immune mechanisms have been implicated to drive this process. CD8^+^ as well as CD4^+^ T cells can mediate lung damage [42–44]. Enhanced innate responses have been reported to contribute to excessive immunopathology following influenza virus infection in pregnant mice [45]. Components of the complement pathway [46] as well as cytokines, such as IL-1 [47], IL-17 [48], and TNF-*α* [49] have been reported to exacerbate pathology in influenza virus-infected lungs.

We assume that, in our study, CD8^+^ T cells that may have exhibited age-related differences in functions within the lung caused the enhanced immunopathology in elderly mice. Impaired functions of antigen-presenting cells during aging have been reported [50, 51], which could in turn alter cytokine production in aged lungs while preserving the T cells' ability to lyse infected epithelial cells. T cell-mediated lysis can cause extensive tissue damage, which has been linked to enhanced immunopathology [52]. In addition, epithelial cell senescence may reduce repair processes following infection-related damages [53], thus further exacerbating morbidity and mortality of aged mice. 

Results obtained with mice do not necessarily translate to humans; nevertheless, data presented here strongly suggest that potent influenza A virus-specific CD8^+^ T cell responses cause fatal immunopathology in aged mice. Taken together, a universal influenza vaccine for aged individuals will need to induce cross-reactive immune responses that are delicately balanced to eliminate the virus without harming an exceedingly vulnerable host.

## 4. Materials and Methods

### 4.1. Ethics Statement

All animal work performed in this study was conducted in accordance with protocols approved by the Wistar Institutional Animal Care and Use Committee; all recombinant DNA work was conducted under the approval of Wistar's Institutional Review Board. 

### 4.2. Mice

Female 6-8-week-old C57Bl/6 mice were purchased from Taconic Labs (Rockville, MD) and kept at the Animal Facility of the Wistar Institute (Philadelphia, PA). All experiments were performed according to institutionally approved protocols. 

### 4.3. Viruses and Vectors

E1-deleted recombinant AdC68 vectors expressing the nucleoprotein of influenza A virus (AdC68NP) or the glycoprotein of rabies virus (AdC68rab.gp) were generated from molecular clones, purified, titrated, and quality controlled as described previously [25, 54]. A/PR8 and A/X31 influenza viruses were grown in the chorioallantoic fluid of embryonated chicken eggs. A/PR8 containing chorioallantoic fluid was titrated by intranasal infection of mice to determine the mean lethal dose (LD_50_). A/X31 virus was titrated on MDCK cells.

### 4.4. Immunization and Infection of Mice

Mice were immunized with the AdC68NP vector, given intramuscularly at 10^10^ vp in 100 *μ*L of sterile PBS. Mice were infected with 0.8–1.6 × 10^5^ TCID50 of A/X31, in 30 *μ*L of sterile PBS, given intranasally to anesthetized mice. Mice were challenged with 3 mean lethal doses (LD_50_) of A/PR8 virus given in 30 *μ*L of saline to the nostrils of sedated mice. Of note in experiments described here naïve mice were used as controls. In preexperiments we assessed if an Ad vector expressing an unrelated antigen, that is, the rabies virus glycoprotein, could influence the outcome of influenza A virus challenges. We observed no statistically significant difference in survival curves or median survival of young or aged mice immunized with the control Ad vector as compared to age-matched naïve mice. 

### 4.5. Isolation of Lymphocytes

Lymphocytes were isolated from blood, spleen, and lung as described previously [25]. In some experiments, samples from the same lungs were used to analyze lymphocytes, virus titers, and histology. In these experiments, the postcaval lobe as used to determine viral titers, the right middle lobe was used to perform histology, and the remaining lobes were used to isolate lymphocytes. Previous experiments were performed testing viral titers from different lobes, as well as all lobes, and results showed no statistical difference. 

### 4.6. Intracellular Cytokine Staining and Tetramer Staining

Intracellular cytokine staining was performed upon stimulation of cells with a peptide carrying the immunodominant H-2^b^ class I binding epitope of NP (aa366–374: ASNENMETM) for 5 hours in presence of Brefeldin as described previously [55]. Antibodies used for ICS were PerCP-Cy5.5-labeled anti-CD8, FITC-labeled anti-IFN-*γ*, APC-labeled anti-IL2, and PE-labeled anti-TNF-*α*. For tetramer staining based on a construct that carried the ASNENMETM peptide, cells were costained with the following additional antibodies: AlexaFlour700-labeled anti-CD44 (BioLegend), PerCP-Cy5.5-labeled anti-CD8, PacificBlue-labeled CD127 (eBioscience), PacificBlue-labeled anti-CD19 (BioLegend), PE-labeled anti-CD160 (Accurate Chemical), FITC-labeled anti-CD62L, FITC-labeled anti-CD94 (BioLegend), FITC-labeled anti-Gr-1 (BioLegend), PE-TexasRed-labeled anti-CD4 (Caltag Labs), PE-Cy7-labeled anti-PD-1 (BioLegend), and PE-Cy7-labeled anti-CD11b (BioLegend). Antibodies to different markers carrying the same dye were tested in separate samples. For flow cytometry, at least 300,000 events were collected for splenocytes and as many events of lymphocyte samples as possible from lungs and blood were acquired on a BD LSR II (BD Biosciences) and analyzed using FlowJo software. 

Lymphocytes were analyzed from individual mice. For phenotypes results from individual mice of a given group were concatenated using FlowJo. In analyses of leukocyte subsets, lymphocytes were first gated on CD4^+^ and CD8^+^ cells, CD4^−^CD8^−^ cells were gated on CD19^+/−^ cells, CD19^−^ cells were gated on Gr-1^+/−^ (or CD94^+/−^cells), and Gr-1^−^ cells (or CD94^−^ cells) were gated on CD11b^+^ cells. 

As described previously for challenge experiments, young and aged naive mice or mice immunized with an Ad vector expressing an unrelated antigen were tested for comparison for T cell responses to NP. Neither showed responses above background (frequencies by tetramer or ICS staining <0.2%).

### 4.7. Virus Titration

Lung virus titers were determined using a TaqMan real-time PCR assay on an ABI Prism 7000 Sequence Detector [27, 56] with influenza A matrix protein gene specific primers. Viral copy numbers were normalized with the original tissue sample masses and calculated based on the molar mass of influenza A/PR8 genome. 

### 4.8. Histology/Pathology

Formalin-fixed lung samples were paraffin-embedded and sectioned at 4 *μ*m for mounting on microscope slides. Section slides of formalin-fixed lung samples were prepared and stained as described. 

### 4.9. Statistical Analyses

Statistical analyses for survival curves were done using GraphPad Prism version 5.00 for Mac, GraphPad Software, San Diego, California, USA, www.graphpad.com. Pairwise group comparisons were done using one-tail, two-sample *t*-tests for comparison of samples with equal variance but different sample sizes using Excel 2008 including Bonferroni correction for multiple testing where specified. Survival analysis was performed using log-rank test. Statistical significance threshold was set at *P* ≤ 0.05 unless stated otherwise.

## Figures and Tables

**Table 1:** Experimental study design.

Groups	Groups of mice (nos. of mice)	Prime	Analyses after the prime (compartment)	Boost (time after prime)	Analyses after the boost (compartment)	Challenge (time after prime)	Time of analyses after challenge (compartment)
Experiment 1 (Figures 1(a) and 1(b))							
Group 1	Young (15)	AdC68NP	Wks 2, 4, 6, and 8 (blood)	None	Wks 2, 4, 6, 8 (blood)	A/PR8 (mo 4)	Day 20 (blood)
Group 2	Aged (13)						
Group 3	Young (4)			A/X31 (mo 2)			
Group 4	Aged (13)						
Group 5	Young (5)	None					
Group 6	Aged (10)						
Group 7	Young (25)			None			
Group 8	Aged (33)						

Experiment 2 (Figures 2(a), 2(b), and 2(c))							
Group 1	Young (5)	AdC68NP		None	Wk 8 (blood, spleen, and lung)	A/PR8 (mo 4)	Days 5, 20 (blood, spleen, and lung)
Group 2	Aged (8)						
Group 3	Young (5)			A/X31 (mo 2)			
Group 4	Aged (5)		None				
Group 5	Young (5)	None					
Group 6	Aged (5)						
Group 7	Young (2)			None			
Group 8	Aged (4)						

Experiment 3 (Figures 3(a) and 3(b))							
Group 1	Young (4)	AdC68NP		None		A/PR8 (mo 4)	Day 20 (spleen)
Group 2	Aged (4)						
Group 3	Young (4)			A/X31 (mo 2)			
Group 4	Young (5)	None	None		None		
Group 5	Aged (4)						
Group 6	Young (5)			None			
Group 7	Aged (3)						

Experiment 4 (Figures 4(a), 4(b), 4(c), and 4(e))^1^							
Group 1	Young (15) [5]	AdC68NP		None		A/PR8 (mo 4)	Checked daily for weight loss and survival; viral titers in lungs tested on day 5; lung infiltrates tested on day 5.
Group 2	Aged (13) [15] [***7***]						
Group 3	Young (4) [5]			A/X31 (mo 2)^2^			
Group 4	Aged (8) [5] [***5***]		None		None		
Group 5	Young (5) [5]	None					
Group 6	Aged (10) [7] [***4***]						
Group 7	Young (25) [9]			None			
Group 8	Aged (38) [11] [***7***]						

^1^Number of mice tested for survival are shown in (), numbers of mice tested for viral loads are shown in regular font in [], and numbers of mice used to determine subsets of lung infiltrating cells are shown on bold italic in [].

^2^Some of the mice were primed at a young age. They were boosted ~18 months later.
